# Caution should be taken when using electronic health record database

**DOI:** 10.1186/s13054-019-2612-5

**Published:** 2019-11-05

**Authors:** Lina Wang, Xiaoming Wu

**Affiliations:** 1 Department of Neurology, Xi’an Ninth Hospital Affiliated to Medical College of Xi’an Jiaotong University, Xi’an, 710054 Shaanxi China; 2 Key Laboratory of Biomedical Information Engineering of Education Ministry, School of Life Science and Technology, Xi’an Jiaotong University, Xi’an, 710049 Shaanxi China

The weekend effect refers [1] to the difference in mortality rate between patients admitted to hospital on weekends and those admitted on weekdays. The weekend effect on patient outcomes has been a practical and research issue. ICU record has been extensively studied in terms of weekend mortality, but the results were not consistent and conclusive [2]. With this as background, Faust et al. [3] recently published an article about the weekend effect on ICU patients in *Critical Care.* The paper is of great interest, and we highly recognized the authors’ logic in the data analysis of this paper.

However, we noted that the data used in the paper might be problematic, and consequently, the results may be unreliable. The cohort data of the paper were from the MIMIC-III database [4], and all admission dates in the database have been shifted to protect patients’ privacy and comply with HIPAA (https://mimic.physionet.org/mimicdata/time/). Admission dates are only internally consistent for the same patient, and this de-identification method altered the real date. Therefore, it is not possible, after alternation, to determine whether a particular admission date was a weekend or not, unless the dates were drifted by adding indefinite integer number of weeks, but that was not mentioned in the description of the MIMIC-III database. One cannot judge whether a specific admission date is a weekend or not because of the drifted admission time in the database. For this reason, we believe that the cohort data in this paper may be inappropriate, and the subsequent results can be unreliable.

In conclusion, we suggest that when using MIMIC-III database for medical analysis, caution should be taken that the dates in the database have been drifted.

## Data Availability

Not applicable.

## Abbreviations

HIPAA: Health Insurance Portability and Accountability Act
ICU: Intensive care unit
MIMIC: Medical Information Mart for Intensive Care
