# Supplementary material for: Caution should be taken when using electronic health record database
Source: Crit Care. 2019 Nov 5;23:345. doi: 10.1186/s13054-019-2612-5 (PMC6829959; doi:10.1186/s13054-019-2612-5)
Supplement: Related texts

# Reply: Authors’ response

**Authors:** Louis Faust, Keith Feldman, Nitesh V. Chawla

We would like to first thank the authors for their concern regarding the reliability of the *day of the week* information in the MIMIC dataset.

While the authors are correct that the true admission dates in MIMIC are shifted, day of the week information has been retained and remains accurate. Unfortunately, note of this retention is absent from the official MIMIC database documentation that the authors (understandably) cite (https://mimic.physionet.org/mimicdata/time/). However, this information can be found on MIT’s official Github, where the MIMIC data repository was utilized as part of the 2018 BHI & BSN Data Challenge (https://github.com/MIT-LCP/bhi-bsn-challenge/blob/master/challenge-demo.ipynb) specifically investigating the “weekend effect.” As stated on the Github page, “A patient-level shift has been applied to dates. Day of week is retained.”

Given the day of the week information is accurate, the results from the manuscript remain reliable.
